# Complete Genome Sequence of Acinetobacter pittii OCU_Ac17, Isolated from Human Venous Blood

**DOI:** 10.1128/MRA.00696-21

**Published:** 2021-09-30

**Authors:** Yuki Matsumoto, Arata Sakiyama, Taishi Tsubouchi, Masato Suzuki, Makoto Niki, Mamiko Niki, Hiroshi Kakeya, Yukihiro Kaneko

**Affiliations:** a Department of Bacteriology, Osaka City University, Graduate School of Medicine, Abeno, Osaka, Japan; b Research Center for Infectious Disease Sciences, Osaka City University, Graduate School of Medicine, Abeno, Osaka, Japan; c Toneyama Institute for Tuberculosis Research, Osaka City University, Graduate School of Medicine, Toneyama, Toyonaka, Japan; d Antimicrobial Resistance Research Center, National Institute of Infectious Diseases, Aoba, Higashi-Murayama, Tokyo, Japan; e Department of Infection Control and Prevention, Osaka City University Hospital, Abeno, Osaka, Japan; University of Maryland School of Medicine

## Abstract

Acinetobacter pittii isolate OCU_Ac17 was obtained from the venous blood of a patient at a hospital in Japan. We present its complete 4.108-Mbp genome sequence (1 chromosome plus 3 plasmids), analyzed by combining long-read (Flongle) and short-read (MiniSeq) sequencing.

## ANNOUNCEMENT

We present the complete genome sequence of Acinetobacter pittii OCU_Ac17, isolated from the venous blood of a patient at Osaka City University Hospital in Osaka, Japan (IRB approval number 3568, 30 September 2016).

OCU_Ac17 was streaked onto blood agar and MacConkey agar after 12.9 h of blood culture at 37°C. It was judged to be an Acinetobacter sp. using MicroScan WorkAway40. It was identified as a strain of A. pittii in the genome analysis performed. A. pittii OCU_Ac17 had been stored at –80°C; it was streaked onto a Mueller-Hinton II agar plate and incubated at 37°C for about 24 h. Genomic DNA (gDNA) extraction was performed using the DNeasy blood and tissue kit (Qiagen). gDNA was sheared to 8-kbp fragments using a g-TUBE device (M&S Instruments Inc., Osaka, Japan). Whole-genome sequencing was performed using the MinION system with a Flongle flow cell (Oxford Nanopore Technologies [ONT]) and the MiniSeq system (Illumina) with a high-output reagent kit (300 cycles). The library for Flongle sequencing was prepared using the ligation sequencing kit (SQK-LSK108) and flow cell (FLO-FLG001), and the library for Illumina sequencing (paired ends; insert size, 500 to 900 bp) was prepared using the Nextera XT DNA library prep kit. The ONT data comprised 159,318 reads (*N*_50_, 7.61 kb) and the Illumina data 3,401,056 reads. In this study, default parameters were used for all bioinformatics softwares except where otherwise noted. The Flongle reads were filtered using Filtlong v0.2.0 (https://github.com/rrwick/Filtlong; length, ≥1,000 bp; quality score, ≥10) to keep only the top 90% of reads, with a target output of 800 Mbp (*N*_50_, 7,847 bp), and the MiniSeq reads were quality filtered using fastp v0.20.1 (1). Both the Flongle and MiniSeq filtered reads were *de novo* assembled using Unicycler v0.4.8 (2), resulting in four circular contigs. A BLAST search for the *rep* gene was conducted among the genes in the DDBJ Fast Annotation and Submission Tool (DFAST), and this gene was manually positioned as the first coding DNA sequence (CDS).

The contigs were polished three times using Pilon v1.24 (3). The complete genome sequence was constructed as a total of 3,835 CDSs, and six sets of rRNA clusters were annotated using the DFAST server (4). The genome lengths and GC contents of the chromosome and each plasmid are shown in Table 1. A BLAST search of six 16S rRNA genes of OCU_Ac17 found that they were related with 99.86 to 99.93% identity to those of A. pittii strain ATCC 19004^T^ (GenBank accession number MN307289.1). Average nucleotide identity (ANI) analysis using pyani v0.2.10 (https://github.com/widdowquinn/pyani) revealed that OCU_Ac17 has 97.82% identity with A. pittii strain ATCC 19004^T^ (GenBank assembly accession number GCA_000369045.1). From these results, OCU_Ac17 was determined to be a strain of A. pittii.

**TABLE 1:** Length and GC content for each plasmid and the chromosome

Element	Length (bp)	GC (%)	No. of reads	DDBJ accession no.
OCUAc17 (complete)	4,108,536	38.8	3,400,945	
OCUAc17 (chromosome)	4,012,621	38.9	3,000,208	AP024798
pOCUAc17-1	69,156	36.7	297,102	AP024799
pOCUAc17-2	15,601	34.4	47,279	AP024800
pOCUAc17-3	11,158	35.1	56,356	AP024801

Multilocus sequence typing (MLST) and detection of antimicrobial resistance genes were performed using MLST v2.0 (5) and ResFinder v4.1 (6), respectively. The results showed that OCU_Ac17 belongs to sequence type 248 (ST248).

### Data availability.

The complete genome sequence of A. pittii isolate OCU_Ac17 has been deposited in DDBJ/ENA/GenBank under accession numbers AP024798, AP024799, AP024800, and AP024801. The raw sequence data are available in the Sequence Read Archive under accession numbers DRX291373 and DRX291374.
